# The efficacy and safety of thermal ablation for patients with lung malignancy: a meta-analysis of 12 studies in China

**DOI:** 10.1186/s13019-022-02090-4

**Published:** 2022-12-22

**Authors:** Rongxing Liu, Qiurong Shen, Hongjun Lu

**Affiliations:** Department of Thoracic and Cardiac Surgery, The Second Hospital of Longyan, Beicheng Shuangyang West Road No. 8, Xinluo District, Longyan, 364000 Fujian China

**Keywords:** Chemotherapy, Thermal ablation, Lung malignancy, Objective response rate, Disease control rate, Progression-free survival, Overall survival, Complication

## Abstract

**Background:**

Thermal ablation has been increasingly used in the treatment of lung cancer in recent years. This meta-analysis aims to investigate the therapeutic effect and safety of thermal ablation plus chemotherapy as compared with chemotherapy alone in treating patients with lung malignancy in China based on current evidence.

**Methods:**

Databases including PubMed, Web of Science, Embase and the Cochrane Library were searched for clinical reports. Additional literature search was also performed by searching the reference list of included studies and latest reviews. Raw data including objective response rate, disease control rate, progression-free survival, overall survival and the incidence of major complication were extracted and pooled.

**Results:**

A total of 12 studies in China including 1282 patients with lung malignancy were included in this meta-analysis. The number of studies that reported data of objective response rate, disease control rate, progression-free survival, overall survival and major complication was 8, 7, 7, 6 and 7, respectively. The combination therapy of thermal ablation plus chemotherapy showed a significantly better efficacy in improving objective response rate (odds ratio = 2.73; *P* < 0.001) and disease control rate (odds ratio = 2.43; *P* < 0.001) as compared with chemotherapy alone. Thermal ablation was also a significant protective factor for progression-free survival (hazard ratio = 0.43; *P* < 0.001) and overall survival (hazard ratio = 0.49; *P* < 0.001). Besides, thermal ablation did not increase the risk of major complication (odds ratio = 0.75; *P* = 0.252).

**Conclusion:**

The present meta-analysis based on these studies in China suggested that thermal ablation is a promising technique to provide better disease response and survival outcomes for patients with lung malignancy. Thermal ablation is worth further promotion in treating lung malignancy and application in clinical practice.

**Supplementary Information:**

The online version contains supplementary material available at 10.1186/s13019-022-02090-4.

## Introduction

Lung malignancy is one of the most hard-to-treat types of cancer in recent years, with the death incidence being the first among all cancer types worldwide [1]. Lung malignancy can be classified into primary diseases including small cell lung cancer (SCLC) and non-small cell lung cancer (NSCLC), and metastatic diseases which most commonly originate from colorectal cancer [2]. Among them, NSCLC is the predominant type which accounts for approximately 80% of total lung cancer cases, and NSCLC is frequently found as adenocarcinoma and squamous cell carcinoma [3].During the recent decades, lung lobectomy has been the first choice in the curative treatment of lung malignancy [4]. However, lung malignancy is difficult to detect and diagnose timely in the early stage due to its mild and obscured symptoms, and it is usually diagnosed when the cancer has progressed. Thus most patients with lung malignancy have missed the best time for surgical resection. Besides, quite a portion of patients are intolerable to major operation due to old age, underling diseases and poor pulmonary function [5]. Patients with unresectable lung malignancy can only receive other treatments such as chemotherapy, EGFR-TKIs. Systemic chemotherapy as the major adjuvant therapy in the treatment of cancer has been widely applied in treating lung cancer. However, the survival benefit after chemotherapy remains limited and poor for patients with unresectable lung malignancy. Given above, a novel therapeutic regimen is necessary to improve the survival outcome of patients with unresectable disease.

Thermal ablation, including radiofrequency ablation (RFA) and microwave ablation (MWA), is an emerging non-operative therapeutic approach with minimally invasiveness. In recent years, thermal ablation has been extensively and successfully applied in the treatment of advanced primary and metastatic cancer which are usually unresectable [6]. The working mechanism of thermal ablation is that tumor tissue turns into necrosis under the local high temperature environment mediated by radiofrequency current or microwave. Thermal ablation is a promising therapy with certain curative potential for lung malignancy. Currently, several studies have explored the efficacy and safety of thermal ablation in combination regimens in treating lung malignancy, and certain significance for short-term efficacy has been observed [7]. However, as an invasive operation, thermal ablation is associated with various complications including pneumothorax, hemorrhage hemoptysis, pneumonia cavitation, etc. Therefore, the use of thermal ablation in treating lung malignancy should be given a further investigation on its effectiveness and safety.

In the present meta-analysis, we focused on investigating the clinical efficacy and safety of thermal ablation plus chemotherapy as compared with chemotherapy alone, and tried to provide a valuable reference for the future treatment of lung malignancy.

## Methods

### Database search

We searched all articles focusing on the combination of thermal ablation plus chemotherapy in patients with either primary or metastatic lung malignancy in the following databases: PubMed, Web of Science, Embase and the Cochrane Library. The databases were searched for studies published from inception to Jan 31, 2022. In PubMED database, we used Medical Subject Headings (MeSH) and text words to generate a three-check subset including thermal ablation, chemotherapy and lung malignancy. The following MeSH terms were used: “radiofrequency ablation” and “microwaves” for identifying the literature of thermal ablation; “chemotherapy, adjuvant” and “drug therapy”, for identifying the literature of chemotherapy; “carcinoma, non-small-cell lung”, “small cell lung cancer” and “lung neoplasms” for identifying the literature of lung malignancy. The following text words including “radiofrequency”, “RFA” and “MWA” were also used for searching literature of thermal ablation. The retrieval formula for this research was generated using “OR” to combine the MeSH terms and text words within each subset, and “AND” to connect the three subsets. Therefore, the generated retrieval formula in PubMed Database was: ((((((radiofrequency ablation[MeSH Terms]) OR (microwaves[MeSH Terms])) OR (radiofrequency[Title/Abstract])) OR (RFA[Title/Abstract])) OR (MWA[Title/Abstract])) AND ((chemotherapy, adjuvant[MeSH Terms]) OR (drug therapy[MeSH Terms]))) AND (((carcinoma, non-small-cell lung[MeSH Terms]) OR (small cell lung cancer[MeSH Terms])) OR (lung neoplasms[MeSH Terms])). The detailed retrieval formula in other databases including Web of Science, Embase and the Cochrane Library was provided in the Additional file 1: Appendix Table.

Additional literature search was performed via examining the reference list of the articles identified and recent reviews. There were two authors assessing the eligibility of literatures for inclusion independently. If there was dissonance of the assessment, further discussion with the third author was conducted to resolve the dissonance. The clinical reports were considered eligible to be included if they fulfilled the following PICOS criteria: (1) P (population): patients with either primary or metastatic lung malignancy; (2) I and C (intervention and comparison): comparative study investigating the combination of thermal ablation plus chemotherapy versus chemotherapy alone; (3) O (outcome): at least one of the following outcomes should be reported: objective response rate (ORR), disease control rate (DCR), progression-free survival (PFS), overall survival (OS) or the number of major complication; (4) S (study design): both retrospective and prospective studies were included. The following articles were excluded during the screening of title, abstract and full text: (1) duplicate records; (2) case report or case series with limited number of patients; (3) specific types of paper without available data such as review, meta-analysis, guideline, letter, comment, editorial, protocol, response, etc.; (4) with less than 10 patients; (5) basic research; (6) no available data were found in the full text review. Endnotes (version X8) was used to manage the articles throughout the literature search and screening process. The protocol of this meta-analysis has been registered in the International Prospective Register of Systematic Reviews (PROSPERO, registration ID: CRD42022307094).

### Data extraction and quality assessment

After the eligible articles were finally included, raw data were extracted by two authors independently. The dissonance of the results was resolved in the similar way as described in the Database Search section. The following characteristics of included studies were collected: year of publication, first author, study location, type of lung malignancy, stage of malignancy, previous treatment, study design, type of ablation (RFA or MWA), total sample size, sample size of the control group (chemotherapy alone) and the experimental group (chemotherapy plus thermal ablation), average age of the sample population, average follow-up time. The following raw statistics were extracted for data synthesis: ORR (alternatively number of complete response (CR) and partial response (PR)), DCR (alternatively CR, PR and stable disease (SD)), HR and 95% CI for PFS and OS, number of major complications in both groups. If Kaplan–Meier curve for PFS and OS were provided instead of HR and 95% CI, then the data of time-to-event were extracted from Kaplan–Meier curve by using the software Engauge, and the data were further used to calculate the HR and 95% CI via the method provided by Tierney et al. [8].

Study quality was accessed based on the coding manual for cohort studies of the Newcastle–Ottawa Scale, which was endorsed by the Cochrane Collaboration to assess the quality of observational studies in its 2011 handbook. The following items were referred for allocation of stars/scores:

1. Representativeness of the exposed cohort;
2. Selection of the non-exposed cohort;
3. Ascertainment of exposure;
4. Demonstration that outcome of interest was not present at start of study;
5. Comparability of cohorts: age;
6. Comparability of cohorts: other factors including gender, race, smoking history, etc.;
7. Assessment of outcome;
8. Was follow-up long enough for outcomes to occur;
9. Adequacy of follow-up of cohorts.

The dissonance of the results was resolved in the similar way as described in the Database Search section. The risk of bias was assessed according to the summary of the above items.

### Definitions

#### ORR and DCR

The evaluation of tumor response was based on the Response Evaluation Criteria in Solid Tumors (RECIST), version 1.1. ORR was defined as the rate of complete response (CR) + partial response (PR), while DCR was defined as the rate of CR + PR + stable disease (SD). Notably, in the experimental group, ORR and DCR should be evaluated after the combination treatments of chemotherapy and ablation, instead of chemotherapy alone. If ORR and DCR in the experimental group were only evaluated for chemotherapy, then the data were considered unsuitable for data synthesis and recorded as NA (not applicable).

#### PFS and OS

PFS was defined as the period from the date of treatment start or the baseline assessment until objective disease progression or subjective disease deterioration or death, whichever occurred first. OS was defined as the time from treatment start or the baseline assessment to the date of death. Progression-free survival was censored on the date of last cancer assessment of patients if the cancer had not progressed. Overall survival was censored on the time of last follow up if patients had not died or lost follow up.

#### Complication

Complication during treatment in both group were assessed based on the Common Terminology Criteria Adverse Events (CTCAE) version 5.0:

1. Grade 1: Mild adverse events (AEs); asymptomatic or mild symptoms; requiring no treatment;
2. Grade 2: Moderate AEs; requiring less treatment; local or non-invasive treatment;
3. Grade 3: Severe AEs but not immediately life-threatening; hospitalization or prolong of hospitalization;
4. Grade 4: Life-threatening; requiring emergency treatment;
5. Grade 5: Death due to AEs.

Major complications were considered as CTCAE grade ≥ 3.

### Main outcomes analysis

Data analysis was performed by two authors independently likewise. As for the mismatch of analysis results calculated by the two authors, a third author would preside over a discussion until consensus was reached. The following outcomes were pooled from raw data: odds ratio (OR) for ORR, DCR and major complication, and HR for PFS and OS.

### Subgroup analysis

Subgroup analysis was conducted via the *metan* module of the STATA software. The studies were divided into subgroups according to the following factors: type of lung cancer, stage of NSCLC, previous treatment (treatment naïve or not), study design (randomized or not), type of ablation (RFA or MWA), total sample size (> or ≤ 100), average age of the sample population (> or ≤ 60), average follow up time (> or ≤ 24 months). Additionally, since HR of PFS and OS was extracted from Kaplan–Meier curve in several studies and there might be subjective bias for this method, we further performed subgroup analysis for PFS and OS in terms of whether HR was extracted from Kaplan–Meier curve.

### Statistical analysis

The risk of bias graph was plotted by using Review Manager Version 5.3 (RevMan, The Cochrane Collaboration, Oxford, United Kingdom). The *metan* module of the STATA software, version 15 (Stata Corporation, College Station, TX) was used to compare the efficacy and safety of chemotherapy plus thermal ablation versus chemotherapy alone. *P*-value < 0.05 was considered as the threshold of statistical significance. Statistical significance (*P* < 0.05) of OR and HR was determined by the Z test. The results were presented as pooled estimate with 95% CI and plotted as forest plot. Heterogeneity of included studies was evaluated via the *I*^2^ statistic and *P* value. A random-effects model was used to pool studies with significant heterogeneity. Sensitivity analysis was conducted by omitting one literature at each analysis to evaluate the effect of each study on the overall result. Publication bias was estimated by using funnel-plot and Egger’s test. The *metaninf* module and *metabias* module of the STATA software were used for sensitivity analysis and publication bias, respectively. The funnel for identifying the underreported articles was also plotted by using the *metafunnel* module of STATA to visually display the results of reporting bias assessment.

All extracted data are summarized in an Excel file which is available in the Additional file 2: Extracted Raw Data.

## Results

### Characteristics of included studies

**Fig. 1:**
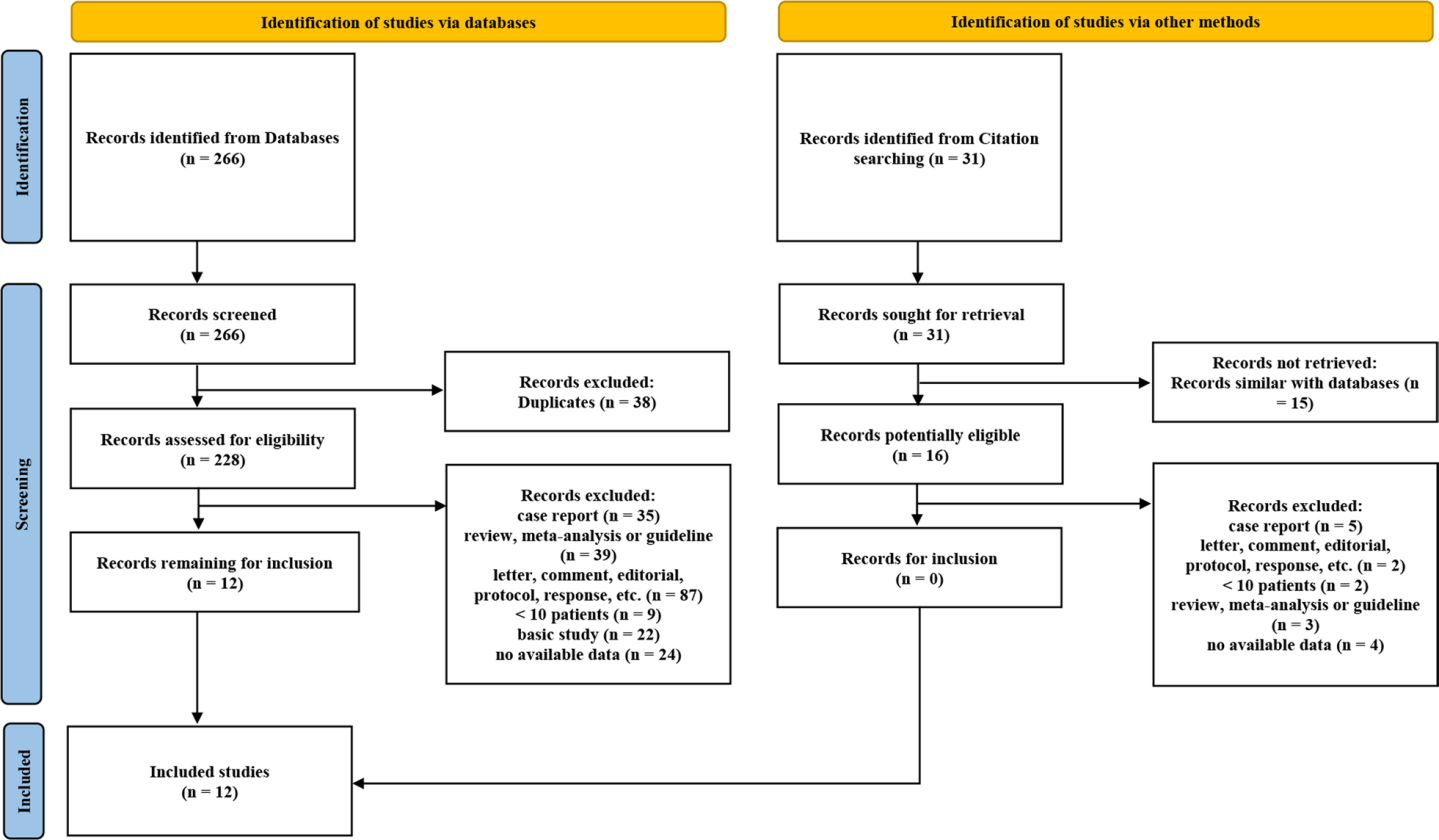
Flow chart of selection of studies

As depicted in Fig. 1, a total of 266 and 31 articles were initially identified from databases searching and citation searching, respectively. After screening by reviewing title, abstract and full text, 12 articles were finally included in the meta-analysis [7, 9–19]. The characteristics of included articles were summarized in Table 1. All the studies were conducted in China, with a total of 1282 patients included. One study included patients with lung metastases from colorectal cancer. Most of the study population were with advanced lung cancer (stage IIIb-IV). Half of the studies were randomized performed. As for the technique of ablation, 5 and 7 studies used RFA and MWA, respectively. The outcomes of patients in each study were listed in Table 2.

**Table 1:** Characteristics of included studies

Year of publication	First author	Study location	Type of lung cancer	Stage of lung cancer	Previous treatment naive?	Randomized?	Type of ablation	Sample size	Average age, year	Average follow up, month
2011	Hua Shen [9]	China	NSCLC	IIIb/IV	No	Yes	RFA	80	59	NA
2014	Sheng Li [10]	China	Metastases	NA	Yes	No	RFA	61	≤ 70	57
2015	Zhigang Wei [11]	China	NSCLC	IIIb/IV	Yes	No	MWA	74	59	21
2016	Zilin Zhao [12]	China	NSCLC	II/IIIa	Yes	Yes	MWA	96	57	39
2016	Shuo Yu [13]	China	NSCLC	III/IV	NA	Yes	RFA	57	58	NA
2019	Wen-Hui Yang [14]	China	NSCLC	IIIb/IV	Yes	No	RFA	93	64	11
2019	Chunhai Li [15]	China	NSCLC	IV	Yes	No	MWA	49	> 60	NA
2020	Zhigang Wei [16]	China	NSCLC	IIIb/IV	Yes	Yes	MWA	293	59	13
2020	Ying-Qing Zhang [17]	China	NSCLC	IIIb/IV	Yes	Yes	MWA	90	69	36
2021	Feng Xu [18]	China	Lung cancer	I-IV	NA	No	RFA	256	48	16
2021	Yuqing Shan [7]	China	NSCLC	IV	Yes	Yes	MWA	67	61	NA
2021	Kan Feng [19]	China	Lung cancer	IIIb/IV	NA	No	MWA	66	47	NA

*NSCLC* non-small cell lung cancer, *NA* not available, *RFA* radiofrequency ablation, *MWA* microwave ablation

**Table 2:** Clinical outcomes of patients included in each study

Study	No. of patients	No. of ORR	No. of DCR	No. of CR	No. of PR	No. of SD	HR of OS	HR of PFS	No. of major complication
*Hua Shen *[9]							NA	NA	
Group C	40	17	36	0	17	19			15
Group A + C	40	19	37	1	18	18			20
*Sheng Li *[10]							0.279	NA	
Group C	22	NA	NA	NA	NA	NA			19
Group A + C	39	NA	NA	NA	NA	NA			26
*Zhigang Wei *[11]							0.59	0.33	
Group C	28	NA	NA	NA	NA	NA			4
Group A + C	46	NA	NA	NA	NA	NA			5
*Zilin Zhao *[12]							NA	NA	
Group C	49	32	34	NA	NA	NA			NA
Group A + C	47	38	41	NA	NA	NA			NA
*Shuo Yu *[13]							NA	NA	
Group C	22	6	16	0	6	10			NA
Group A + C	35	28	33	0	28	5			NA
*Wen-Hui Yang *[14]							0.88	0.92	
Group C	45	15	30	0	15	15			24
Group A + C	48	18	34	0	18	16			15
*Chunhai Li *[15]							NA	0.153	
Group C	28	NA	NA	NA	NA	NA			19
Group A + C	21	NA	NA	NA	NA	NA			16
*Zhigang Wei *[16]							0.38	0.44	
Group C	145	NA	NA	NA	NA	NA			93
Group A + C	148	NA	NA	NA	NA	NA			84
*Ying-Qing Zhang *[17]							NA	NA	
Group C	42	4	26	0	4	22			NA
Group A + C	48	16	42	0	16	26			NA
*Feng Xu *[18]							0.47	0.5	
Group C	128	57	87	19	38	30			NA
Group A + C	128	79	106	28	51	27			NA
*Yuqing Shan *[7]							NA	0.42	
Group C	33	12	NA	0	12	NA			NA
Group A + C	34	24	NA	8	16	NA			NA
*Kan Feng *[19]							0.49	0.379	
Group C	33	8	20	3	5	12			0
Group A + C	33	21	28	10	11	7			0

*Group C* group chemotherapy, *Group A* + *C* group ablation + chemotherapy, *ORR* objective response rate, *DCR* disease control rate, *CR* complete response, *PR* partial response, *SD* stable disease, *HR* hazard ratio, *OS* overall survival, *PFS* progression-free survival, *NA* not available

**Fig. 2:**
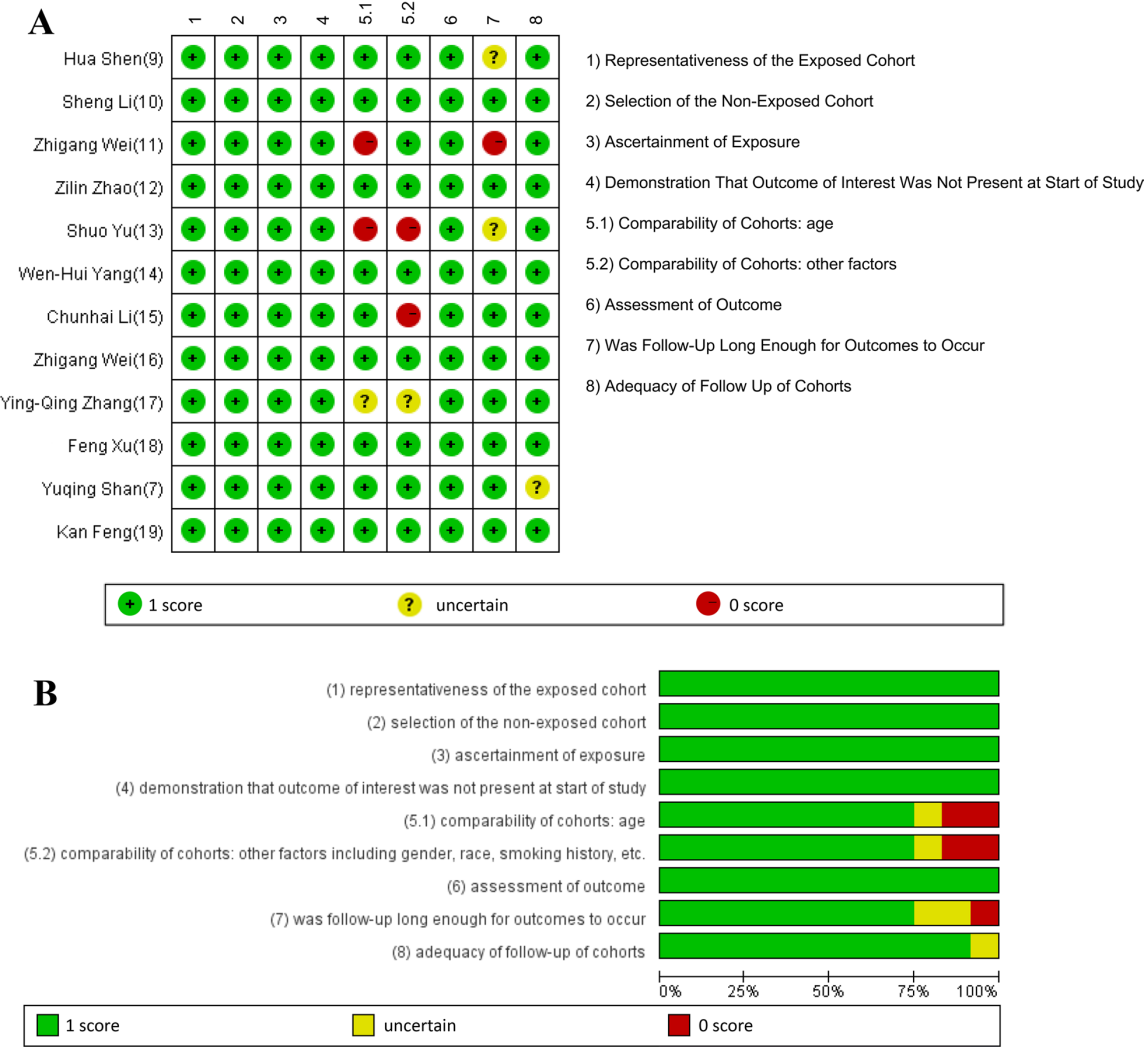
Quality assessment of included studies: **A** the figure shows the authors' judgments about each aspect of quality item for each included study. **B** The results are also presented as percentages across all included studies

As depicted in Fig. 2A and B, 11 of the 12 studies were considered as high quality with NOS score ≥ 7. All studies had good performance in “representativeness of the exposed cohort”, “selection of the non-exposed cohort”, “ascertainment of exposure”, “demonstration that outcome of interest was not present at start of study and assessment of outcome”. While for the “comparability of cohorts: age”, “comparability of cohorts: other factors” and “was follow-up long enough for outcomes to occur”, 9 of 12 studies got 1 score.

### Main finding: ORR

**Fig. 3:**
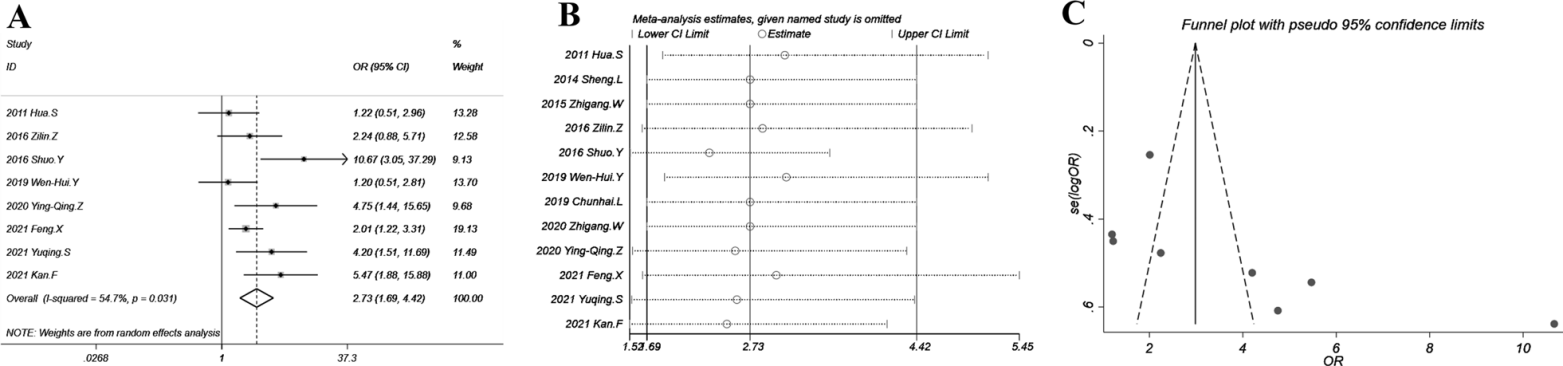
Objective response rate of chemotherapy plus thermal ablation versus chemotherapy alone: **A** forest plot for overall odds ratio; **B** sensitivity analysis; **C** funnel plot for publication bias evaluation

The combination therapy induced better ORR with OR = 2.73 (95% CI 1.69–4.42; *P* < 0.001). The forest plot in Fig. 3A showed that all included studies had an OR > 1. The heterogeneity was moderate with *I*^2^ = 55% and *P* = 0.031. Sensitivity analysis in Fig. 3B suggested a relatively stable result with all ORs and 95% CIs > 1. The ORs ranged from 2.5 to 3.08. The funnel plot (Fig. 3C) showed a slight asymmetry with the study by Shuo et al*.* deviating from the funnel, although Egger’s test suggested no significant publication bias (*P* = 0.084). The former sensitivity analysis showed that omitting the study by Shuo et al*.* yielded an OR of 2.32 with 95% CI of 1.53–3.54 which was not significantly different with the overall OR. Thus the effect of potential publication bias was quite minor.

### Main finding: DCR

**Fig. 4:**
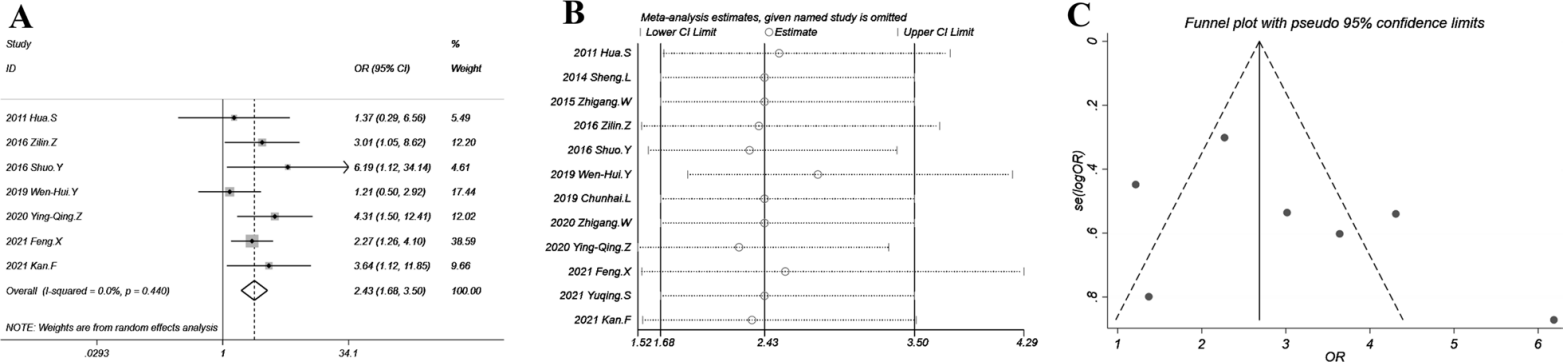
Disease control rate of chemotherapy plus thermal ablation versus chemotherapy alone: **A** forest plot for overall odds ratio; **B** sensitivity analysis; **C** funnel plot for publication bias evaluation

The combination therapy of chemotherapy plus thermal ablation significantly improved the disease control rate with OR = 2.43 (95% CI 1.68–3.5; *P* < 0.001). The forest plot in Fig. 4A showed that all included studies had an OR > 1. There was no obvious heterogeneity with *I*^2^ = 0% and *P* = 0.44. Sensitivity analysis in Fig. 4B showed a very stable result with ORs ranging from 2.31 to 2.81 and all 95% CIs > 1. Egger’s test indicated no significant publication bias (*P* = 0.251). Similarly, the study by Shuo et al*.* deviating from the funnel led to slight asymmetry of the funnel plot (Fig. 4C). Omitting the study by Shuo et al*.* yielded an OR of 2.32 with 95% CI of 1.59–3.38 which was not different with the overall OR. Therefore the impact of potential publication bias was also minimal.

### Main finding: PFS

**Fig. 5:**
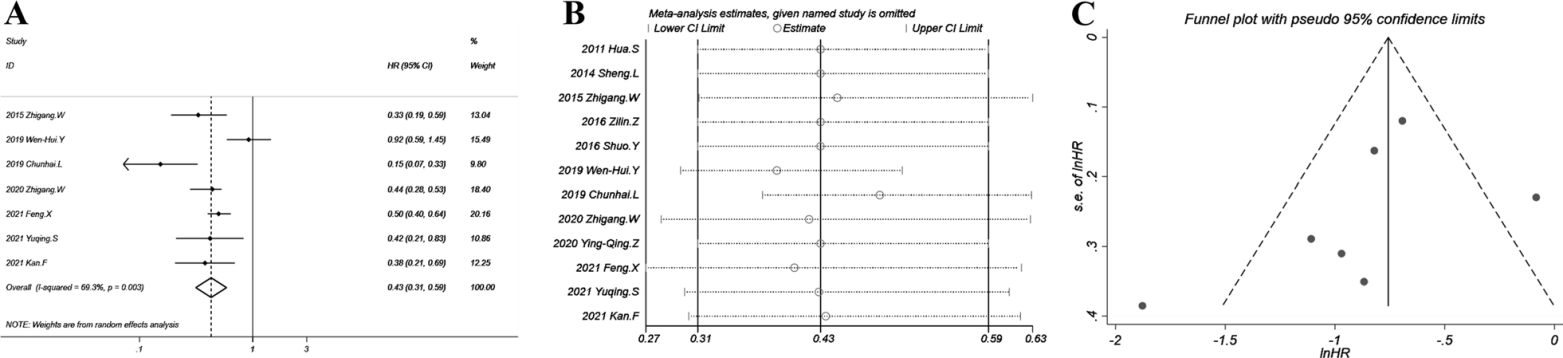
Progression-free survival of chemotherapy plus thermal ablation versus chemotherapy alone: **A** forest plot for overall odds ratio; **B** sensitivity analysis; **C** funnel plot for publication bias evaluation

The pooled HR (0.43; 95% CI 0.31–0.59; *P* < 0.001) indicated that the combination of chemotherapy and thermal ablation could more effectively protect patients from disease progression as compared with chemotherapy alone (Fig. 5A). The heterogeneity was moderate with *I*^2^ = 69% and *P* = 0.003. Sensitivity analysis in Fig. 5B showed that HRs ranged from 0.39 to 0.49 and all 95% CIs < 1, which were relatively stable with only minor variations as compared with the overall HR (0.43). Egger’s test indicated no significant publication bias (*P* = 0. 331). The funnel plot in Fig. 5C was basically symmetrical.

### Main finding: OS

**Fig. 6:**
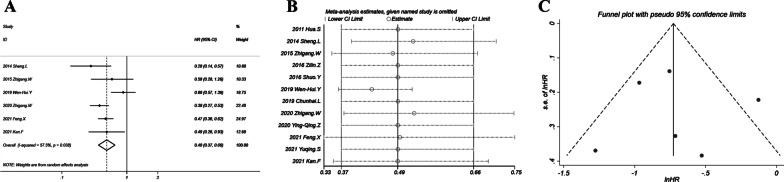
Overall survival of chemotherapy plus thermal ablation versus chemotherapy alone: **A** forest plot for overall odds ratio; **B** sensitivity analysis; **C** funnel plot for publication bias evaluation

The combination therapy of thermal ablation plus chemotherapy significantly improved the overall survival with HR = 0.49 (95% CI 0.37–0.66; *P* < 0.001) (Fig. 6A). There was moderate heterogeneity with *I*^2^ = 58% and *P* = 0.038. The HRs in the sensitivity analysis in Fig. 6B ranged from 0.43 to 0.53 with all 95% CIs < 1. Egger’s test indicated no significant publication bias (*P* = 0.92) and the funnel plot in Fig. 6C showed good symmetry.

### Main finding: major complication

**Fig. 7:**
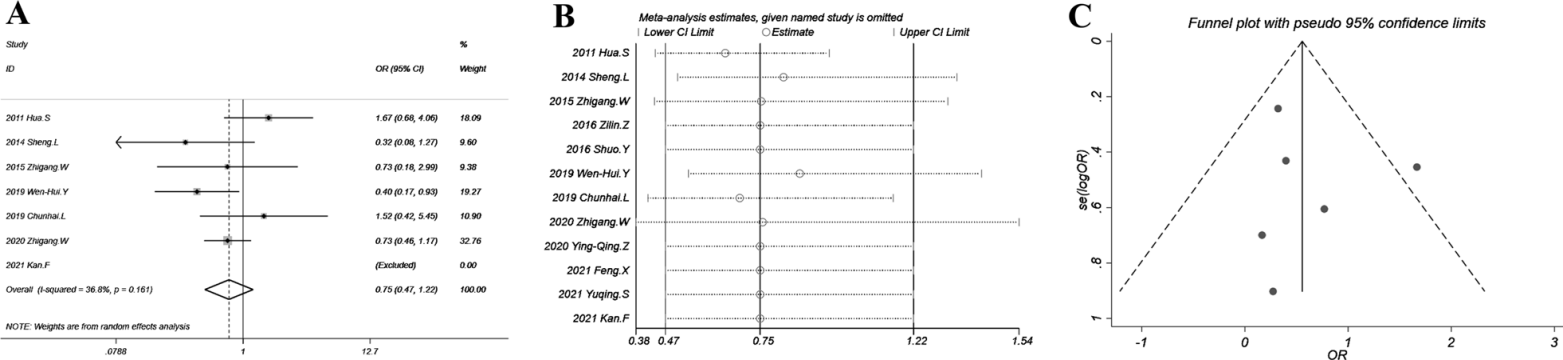
Major complication of chemotherapy plus thermal ablation versus chemotherapy alone: **A** forest plot for overall odds ratio; **B** sensitivity analysis; **C** funnel plot for publication bias evaluation

As shown in Fig. 7A, the risk of major complication of the combination treatment and chemotherapy monotherapy was not different (OR = 0.75; 95% CI 0.47–1.22; *P* = 0.252). The heterogeneity was minor with *I*^2^ = 37% and *P* = 0.161).The sensitivity analysis also supported the main finding, with lower limits of 95% CIs < 1 and upper limits > 1 (Fig. 7B). Egger’s test indicated no significant publication bias (*P* = 0.686) and the funnel plot in Fig. 7C showed good symmetry.

### Additional analysis: subgroup analysis

The subgroup analysis further supported the main findings of the meta-analysis, with significantly improved ORR, DCR, PFS and OS in almost all subgroups with number of studies > 1 (Table 3). Notably, despite that there might be subjective bias for HRs of PFS and OS extracted from Kaplan–Meier curve, it turned out that the survival results remained the same regardless of whether HR was extracted from Kaplan–Meier curve. As for the heterogeneity, heterogeneity was not significantly reduced by dividing the studies into different subgroups according to the above mentioned variables.

**Table 3:** Subgroup analysis

Subgroup	ORR			DCR			PFS			OS		
	No. of studies	OR and P	*I*^2^ (%)	No. of studies	OR and P	*I*^2^ (%)	No. of studies	HR and P	*I*^2^ (%)	No. of studies	HR and P	*I*^2^ (%)
*Type of lung cancer*												
NSCLC	6	2.73; 0.003	60.5	5	2.44; 0.004	25.1	5	0.4; 0.001	78.7	3	0.57; 0.064	77.9
Lung cancer	2	2.95; 0.026	64.1	2	2.49; 0.001	0.0	2	0.48; 0.001	0.0	2	0.47; 0.001	0.0
Metastases	0	NA	NA	0	NA	NA	0	NA	NA	1	0.27; 0.001	0.0
*Stage of lung cancer*												
Advanced	6	3.21; 0.001	65.4	5	2.54; 0.004	29.1	6	0.4; 0.001	73.8	4	0.55; 0.009	67.0
Other	2	2.05; 0.001	0.0	2	2.43; 0.001	0.0	1	0.5; 0.001	0.0	2	0.4; 0.001	42.8
*Previous treatment naive?*												
Yes	4	2.51; 0.005	39.7	3	2.38; 0.027	45.0	5	0.4; 0.001	78.7	4	0.49; 0.008	74.4
No	1	1.22; 0.653	0.0	1	1.37; 0.693	0.0	0	NA	NA	0	NA	NA
NA	3	4.33; 0.005	73.7	3	2.7; 0.001	0.0	2	0.48; 0.001	0.0	2	0.47; 0.001	0.0
*Randomized?*												
Yes	5	3.29; 0.001	56.9	4	3.31; 0.001	0.0	2	0.43; 0.001	0.0	1	0.38; 0.001	0.0
No	3	2.19; 0.028	58.1	3	2.05; 0.006	16.3	5	0.41; 0.001	79.2	5	0.52; 0.001	56.2
*Type of ablation*												
RFA	4	2.11; 0.045	68.1	4	1.96; 0.008	9.6	2	0.65; 0.166	82.0	3	0.51; 0.017	77.8
MWA	4	3.76; 0.000	0.0	3	3.61; 0.001	0.0	5	0.34; 0.001	40.1	3	0.42; 0.001	0.0
*Average age, year*												
≤ 60	5	2.81; 0.001	61.9	5	2.61; 0.001	0.0	4	0.45; 0.001	0.0	4	0.44; 0.001	0.0
> 60	3	2.7; 0.034	59.6	2	2.2; 0.211	69.3	3	0.4; 0.086	88.0	2	0.51; 0.246	85.9
*Average follow up, month*												
> 24	2	2.98; 0.004	0.0	2	3.59; 0.001	0.0	4	0.51; 0.001	69.2	1	0.27; 0.001	0.0
≤ 24	2	1.75; 0.014	4.4	2	1.81; 0.048	25.6	0	NA	NA	4	0.53; 0.001	68.2
NA	4	3.91; 0.004	67.7	3	3.14; 0.007	0.0	3	0.29; 0.001	55.8	1	0.49; 0.029	0.0
*HR extracted from KM curve?*												
Yes	NA	NA	NA	NA	NA	NA	4	0.51; 0.001	67.1	3	0.38; 0.001	0.0
No	NA	NA	NA	NA	NA	NA	3	0.31; 0.001	68.7	3	0.61; 0.031	65.3

*ORR* objective response rate, *DCR* disease control rate, *PFS* progression-free survival, *OS* overall survival, *OR* odds ratio, *HR* hazard ratio, *NSCLC* non-small cell lung cancer, *NA* not available, *RFA* radiofrequency ablation, *MWA* microwave ablation

## Discussion

The main findings in the present meta-analysis suggest that thermal ablation can significantly improve the disease response and survival outcomes for patients with lung malignancy. Out of our expectation, as an invasive operation, thermal ablation did not significantly increase the risk of major complication. Actually, most of the studies suggested that the combination of thermal ablation and chemotherapy might reduce the probability of major complication. For instance, in the study by Yang et al*.*, the rate of major complication of combination group was 31.3% (n = 48) while in chemotherapy group 53.3% (n = 45) [14]. Thermal ablation significantly reduced the incidences of weakness (12.5% versus 46.7%, *P* < 0.001) and gastrointestinal reaction (25.0% versus 77.8%, *P* < 0.001) of patients undergoing chemotherapy. A reasonable explanation is that thermal ablation can substantially alleviate the tumor burden so as to reduce the probability of complication. Another explanation by the authors is that hyperthermia as a physical therapy could help relax patients and enhance metabolism, which might be associated with the potential inhibition of the secretion of 5-hydroxytryptamine and neurokinin-1. According to Wei et al. [16] ablation-related major complications only account for approximately 20% in the MWA plus chemotherapy group. It seems that the benefit of ablation has overwhelmed its invasiveness. There are several advantages of thermal ablation in treating primary and metastatic lung malignancy. The ideal treatment outcome for wedge resection, lobar resection and pneumonectomy would be to eliminate the malignancy with minimal loss of lung parenchyma [20]. In contrast, image-guided thermal ablation aims to induce cancer tissue necrosis precisely which is not limited by the segmental anatomy of the lungs, thus more pulmonary tissue peripheral to the tumor can be preserved [21]. More importantly, the treatment of thermal ablation leaves room for further repeated ablations, considering the multiple lesions of advanced lung malignancy as well as the frequent recurrence [22]. The minimal invasiveness is another advantage of thermal ablation. Pulmonary ablation can be commonly well tolerated by the majority of patients, even those with reduced cardiac and pulmonary reserve. According to a retrospective single-institution study of 1000 RFA sessions in 420 patients by Masataka et al. [23], the procedure-related mortality was 0.4% with one death from hemothorax and three deaths from interstitial pneumonia. In the current meta-analysis, the superiority of thermal ablation has been reflected by the significantly improved treatment efficacy without increased major complications, which can be owed to the above features of thermal ablation.

The subgroup analysis in this meta-analysis suggests that both RFA and MWA can enhance the treatment efficacy. Currently, there is limited data on comparing the efficacy of RFA and MWA in treating lung malignancy. There are also no relevant guidelines or expert consensus concerning the optimal selection of ablative technique in treating lung malignancy. The lung has higher electric impedance than the liver and kidney due to its aerated feature, as the air in the lung tissue is a great insulator for thermal energy. Therefore, MWA might be more effective in treating lung malignancy since it can create a more predictable, confluent, and larger necrotic volume which is less susceptible to heat sink effect due to its higher electromagnetic frequency [24, 25]. MWA can also sufficiently induce one pulmonary lesion within only five minute after antenna placement, which is much faster than RFA [26]. However, MWA might lead to increased complication due to its greater power. The antenna of MWA should usually be placed 2 cm of aerated lung between the ablation zone and the pleural surface if possible so as to reduce the probability of prolonged pain, pneumothorax, and skin burn [27]. Future clinical investigations comparing the ablation modalities may be useful in the treatment strategy for patients with lung malignancy.

There is heterogeneity for several analysis in this study. For instance, there is heterogeneity with *I*^2^ = 55% for the analysis of ORR. According to the Cochrane Handbook Version 5.1.0 of systematic review, the heterogeneity should be considered as “moderate”. The source of heterogeneity should be due to the intrinsic characteristics of the current study design, which included both prospective and retrospective studies. The importance of the observed value of *I*^2^ (55%, moderate) depends on the magnitude and direction of effects. In the forest plot of Fig. 3A, we observed that all included studies had an OR > 1. The lower limit of 95% CI more than a half of the studies (5 of 8) was also larger than 1. Therefore these data strongly suggested that the combination therapy improved objective response rate. Besides, sensitivity analysis in Fig. 3B also indicated a relatively stable result with all ORs and lower 95% CI limits > 1, which further confirmed the efficacy of combination therapy. Based on these analysis, the test efficacy should be considered as sufficient and convincing.

There are several limitations of this meta-analysis. First, the size of population in each study included is relative small. This is a inherent limitation for current available studies since thermal ablation has been used in patients with lung malignancy only in recent years. Second, there exists heterogeneity of included studies, and the source of heterogeneity has not been fully determined in the current meta-analysis. We considered that the different study design in each study might lead to greater deviation of the outcomes, thus enhancing the overall heterogeneity. Third, the articles included in this study are all within the scope of China. We aimed to search the studies worldwide at the first stage of this meta-analysis. However, only studies in China were finally identified and eligible for inclusion. This might be due to that the current thermal ablation in treating lung malignancy is mostly applied in Chinese medical centers. Besides, there are a large number of patients with lung malignancy in China. Thus currently only studies in China with ample patients are qualified to be included in this meta-analysis.

## Conclusion

Thermal ablation is an effective, well-tolerated and safe local treatment method for lung malignancy. These studies in China suggested that thermal ablation is a promising technique to improve the tumor response and patient survival for patients with lung malignancy undergoing chemotherapy. This meta-analysis provides supporting evidence for the clinical application of thermal ablation in treating lung malignancy.

## Supplementary Information

**Additional file 1. Appendix table**. The additional WORD file lists the keywords used in this study and detailed retrieval formula in databases.

**Additional file 2. Extracted Raw Data**. The additional EXCEL file shows all the raw data extracted from the included articles and used for data analysis.

## Data Availability

The authors confirm that the data supporting the findings of this study are available within the article and its Additional files 1, 2.

## Abbreviations

NSCLC: Non-small cell lung cancer
SCLC: Small cell lung cancer
RFA: Radiofrequency ablation
MWA: Microwave ablation
ORR: Objective response rate
DCR: Disease control rate
PFS: Progression-free survival
OS: Overall survival
CR: Complete response
PR: Partial response
SD: Stable disease
OR: Odds ratio
HR: Hazard ratio
CTCAE: Common Terminology Criteria Adverse Events
AE: Adverse event
NA: Not available
