# Supplementary material for: The efficacy and safety of thermal ablation for patients with lung malignancy: a meta-analysis of 12 studies in China
Source: J Cardiothorac Surg. 2022 Dec 22;17:334. doi: 10.1186/s13019-022-02090-4 (PMC9784073; doi:10.1186/s13019-022-02090-4)
Supplement: Supplementary file 1 — Additional file 1. Appendix table. The additional WORD file lists the keywords used in this study and detailed retrieval formula in databases. [file 13019_2022_2090_MOESM1_ESM.docx]

**Retrieval formula in Web of Science:**

#1: TS=(radiofrequency ablation) OR TS=(microwave ablation) OR TS=(RFA) OR TS=(MWA) OR TS=(ablation)

#2: TI=(chemotherapy) OR TI=(pemetrexed) OR TI=(platinum) OR TI=(carboplatin) OR TI=(paclitaxel)

#3: TS=(lung cancer) OR TS=(lung malignancy) OR TS= (NSCLC) OR TS= (non small cell lung cancer)

#4: #1 AND #2 AND #3

**Retrieval formula in Embase:**

#1: ('radiofrequency ablation'):ti OR ((microwave):ti) OR ((RFA):ti) OR ((MWA):ti) OR ((ablation):ti)

#2: (chemotherapy):ti OR ((pemetrexed):ti) OR ((platinum):ti) OR ((carboplatin):ti) OR ((paclitaxel):ti)

#3: ('lung cancer'):ti OR (('lung malignancy'):ti) OR ((NSCLC):ti) OR (('non small cell lung cancer'):ti)

#4: #1 AND #2 AND #3

**Retrieval formula in the Cochrane Library:**

#1: (radiofrequency ablation):ti,ab,kw OR (microwave):ti,ab,kw OR (RFA):ti,ab,kw OR (MWA):ti,ab,kw AND (ablation):ti,ab,kw

#2: (chemotherapy):ti,ab,kw OR (pemetrexed):ti,ab,kw OR (platinum):ti,ab,kw OR (carboplatin):ti,ab,kw AND (paclitaxel):ti,ab,kw

#3: (lung neoplasms):ti,ab,kw OR (lung cancer):ti,ab,kw OR (lung malignancy):ti,ab,kw OR (NSCLC):ti,ab,kw AND (non small cell lung cancer):ti,ab,kw

#4: #1 AND #2 AND #3
